# Harnessing Population Genomics, Gut Microbiota, and Environmental DNA Surveillance for the Conservation of Chinese Spotted Seals in a Changing World

**DOI:** 10.1002/ece3.72952

**Published:** 2026-02-03

**Authors:** Shuzhen Li, Wenzhuo Li, Xuwang Zhang, Hao Zhou, Jingjing Zhan

**Affiliations:** ^1^ Key Laboratory of Industrial Ecology and Environmental Engineering (Ministry of Education), School of Chemical Engineering, Ocean and Life Sciences Dalian University of Technology Panjin China

**Keywords:** environmental DNA, genetic diversity, gut microbiome, marine ecosystem, metagenomics, molecular biology

## Abstract

The triple planetary crisis—encompassing climate change, biodiversity loss, and pollution—poses escalating threats to Earth's systems, particularly impacting marine mammals. The spotted seal ( *Phoca largha* Pallas 1811), currently recognized as the only pinniped species known to breed in China, holds the status of a National Grade I protected species in China. To elucidate the genetic diversity of Chinese spotted seal populations and provide scientific foundations for their conservation and management, this review systematically summarized the fundamental biological characteristics and documented migration routes of spotted seal populations in China, with particular emphasis on reviewing molecular‐level research advancements regarding population genetic structure. Early studies primarily employed molecular markers such as microsatellite DNA and mitochondrial DNA (mtDNA), revealing relatively low genetic diversity levels within Chinese spotted seal populations. In recent years, rapid developments in omics technologies have enabled comprehensive investigations into both genomic compositions, as well as gut microbial community diversity and functional profiles of this species. Furthermore, this review critically examined current research limitations and challenges while proposing the potential advantages and developmental trends of environmental DNA (eDNA) technology in future population studies. These technological and strategic advancements are anticipated to significantly enhance survey efficiency and conservation effectiveness for Chinese spotted seal populations.

## Graphical abstract

Marine mammals are sentinel species for ocean and human health. Multiple anthropogenic and environmental stressors threaten these organisms, particularly China's spotted seals, which have experienced significant decline over the past half‐century. Our review reveals low genetic diversity in Chinese spotted seal populations. Recent gut microbiota studies are vigorously pursued, revealing close host‐microbiota‐health interlinks. This synthesis examines current research limitations while proposing environmental DNA technology's advantages and developmental trends for future studies.

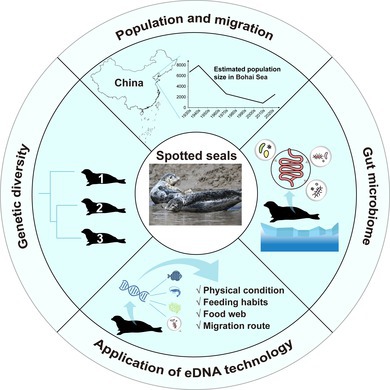

## 1 Introduction

The spotted seal, also known as the Largha sea, belongs to the class Mammalia, order Carnivora, suborder Caniformia, family Phocidae, and genus *Phoca*. As a marine mammal inhabiting temperate and subarctic zones, it is a shared species among the coastal waters of China, North Korea, and South Korea. Its distribution spans the northern and western regions of the North Pacific Ocean, including the Beaufort Sea, Chukchi Sea, Sea of Japan, Bering Sea, Okhotsk Sea, and China's Yellow Sea and Bohai Sea. Notably, the Liaodong Bay in the Bohai Sea serves as the sole breeding ground for spotted seals in China, situated at the southernmost among the eight recognized global breeding sites for spotted seals. As a piscivorous marine predator, the spotted seal primarily feeds on fish, with an adult weighing approximately 70 kg requiring a daily intake of 7–8 kg of fresh fish. Its diet also includes crustaceans and cephalopods (Han 2009). Functioning as an apex predator in the Yellow and Bohai Seas ecosystem, the spotted seal holds significant value across multiple domains, including economic development (e.g., fisheries interactions), ecotourism, medical research (e.g., physiological adaptations to marine environments), ecological balance maintenance, and scientific studies on Arctic‐migratory marine mammals.

The triple planetary crisis (i.e., climate change, biodiversity loss, and pollution) fundamentally destabilizes marine ecosystems, with marine mammals facing disproportionate vulnerability due to their apex trophic positions and long generation times (Hellweg et al. 2023). Ocean warming driven by climate change poses multifaceted threats to marine mammals, altering their habitats through rising sea levels, diminished sea ice, and salinity shifts (Harvell et al. 2002). These environmental disruptions force distributional changes that fragment populations, trigger prey‐phenology mismatches, and critically endanger migratory species reliant on seasonal resource pulses (Albouy et al. 2020). Elevated temperatures directly compromise survival by increasing physiological stress, expanding pathogen transmission, and reducing reproductive success. Such impacts disproportionately threaten the biodiversity of marine mammals, as 37% of them are considered endangered by the IUCN (Davidson et al. 2012).

Additionally, recent studies have confirmed the pervasive accumulation of multiple anthropogenic pollutants in spotted seals. Laser direct infrared imaging spectroscopy (LDIR) analysis of fecal samples from Bohai populations detected microplastics (MPs) including polyethylene, polypropylene, and polystyrene polymers (Pan et al. 2025). Critically, persistent MPs detection in stomach contents since 2012 across the Bering–Chukchi Seas indicates pan‐Arctic contamination (Sletten et al. 2025). Concurrently, heavy metals including Hg, Cd, Pb, As, Fe, and Mn were identified in seal feces, exhibiting concentrations comparable to those in Liaodong Bay fish, demonstrating trophic transfer (Han 2009). Furthermore, elevated petroleum hydrocarbons (~11.1 mg/kg) in fish suggest potential bioaccumulation amplification in seals (Wang et al. 2014). This contaminant triad (MPs‐metals‐hydrocarbons) threatens population resistance and resilience through metabolic disruption and generational impacts (Schaap et al. 2023; Zantis et al. 2021), further establishing seals as critical sentinel species for monitoring marine pollution gradients across ecosystems (Bossart 2011).

In this review, we examine the current state of Chinese spotted seal research, with a particular focus on molecular approaches for characterizing population structure. First, we assess how global change drivers are reshaping the species' population dynamics and elucidate its complex migratory pathways. Second, we synthesize molecular studies on population genetics to decode evolutionary adaptations. Third, we outline gut microbiota diversity and identify four key research priorities, revealing mechanistic associations between the gut microbiome and host health status. Finally, we propose future research directions and strategies based on environmental DNA (eDNA) technology, providing a foundation for spotted seal conservation under accelerating global change.

## 2 Population Variations and Migration Patterns

**FIGURE 1:**
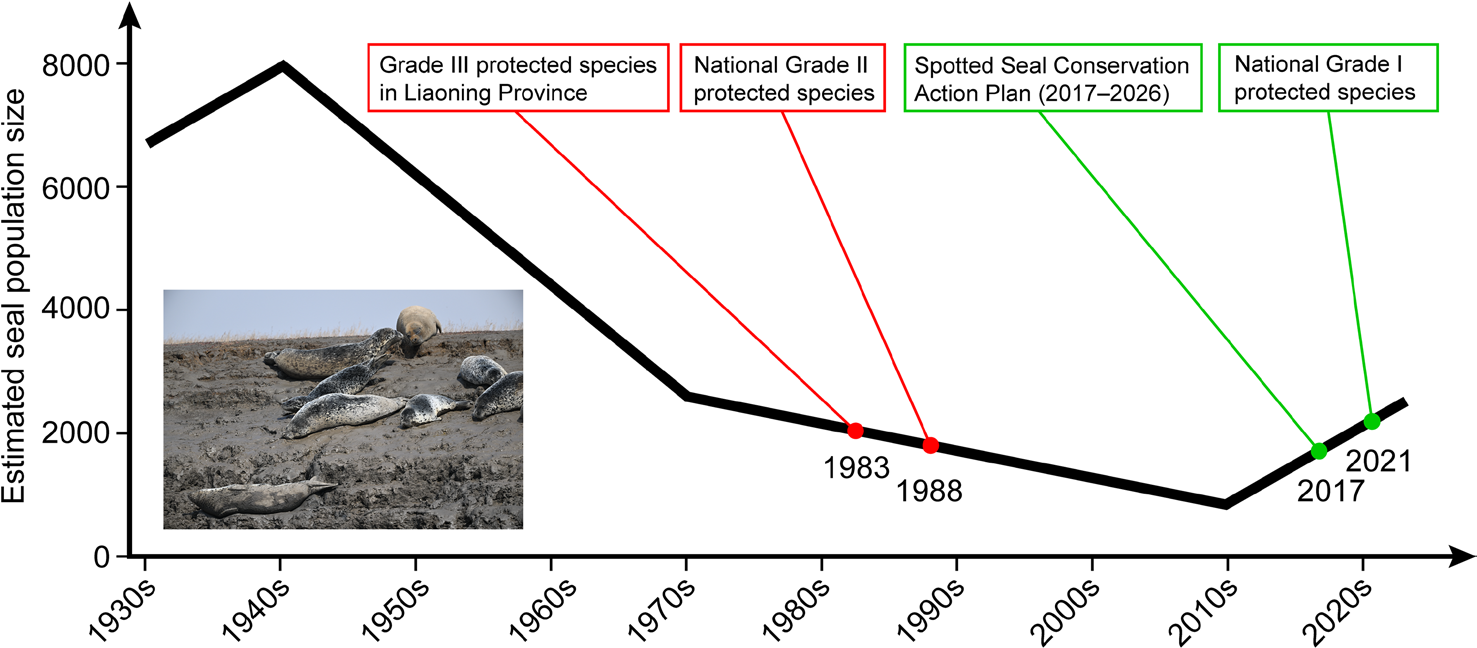
Estimated numbers of spotted seals in the Bohai Sea alongside key national protection policies. Population data are compiled from historical records and published surveys, derived from multiple methods including fishing catch records and aerial/vessel‐based observations.

The spotted seal exhibits extensive migratory ranges and demonstrates high sensitivity to environmental disturbances, frequently altering its migration routes in response to anthropogenic interference. This behavioral plasticity complicates accurate population census and migration tracking (Wang and Ding 2019). Historically, population estimates were derived from fishing catch records combined with field observations. Such methodology suggested approximately 8000 individuals inhabited Liaodong Bay during 1930–1940 (Figure 1). Following China's implementation of hunting prohibitions in the 1980s, subsequent surveys employed aerial and vessel‐based observations using binoculars and photographic documentation (P. Wang 1988). Over the past half‐century, severe habitat degradation caused by overhunting, wastewater discharge, petroleum extraction, port construction, and maritime transportation precipitated an 80% population decline in the Bohai Sea subpopulation. Estimates from fishing vessel observations in 1982 indicated fewer than 2000 individuals, while aerial surveys around 2007 estimated approximately 1000 remaining individuals. A long‐term vessel‐based monitoring around 2015 revealed a partial recovery to roughly 2000 individuals (Wang et al. 2008; Wang and Ding 2019).

Global awakening to biodiversity conservation and marine ecosystem protection has driven substantial improvements in habitat quality through enhanced environmental governance, species‐specific legislation, and scientifically designed protected areas. These interventions have facilitated significant population recovery. The spotted seal received progressive conservation status upgrades in China, being designated as a Grade III protected species by Liaoning Province in 1983, elevated to National Grade II protected species in 1988, and ultimately granted National Grade I protected species status in 2021 (Qu et al. 2021). China has established several critical conservation areas, such as Dalian and Panjin in Liaoning Province, and Miaodao in Shandong Province. In 2017, the Ministry of Agriculture of the PRC issued the Spotted Seal Conservation Action Plan (2017–2026), which systematically intensified research and habitat conservation efforts through coordinated policy implementation and scientific management protocols. Furthermore, *ex situ* conservation through controlled captive breeding has emerged as a critical strategy, with China's captive population expanding from 400 individuals in 2012 to over 1000 currently (Tian, Wang, et al. 2022). This approach complements in situ efforts, providing valuable insights into reproductive biology while maintaining genetic reservoirs for potential reintroduction programs.

The spotted seal exhibits remarkable ecological adaptability, thriving across a broad spectrum of water temperatures (−12°C to 33°C) and salinities, capable of inhabiting both freshwater and marine environments (Han 2009). Annually, populations migrate into the Bohai Sea between November and December, with parturition occurring on drifting ice floes from January to March of the following year. Neonates undergo a lactation period of approximately 1 month, while adults initiate reproductive activities prior to weaning, followed by an 11‐month gestation period. By late May, most individuals depart the Bohai Sea for other marine regions. In general, migration patterns of the spotted seal have been primarily investigated through satellite telemetry tracking techniques. Tracking data from 23 individuals reintroduced into the wild between 2010 and 2020 revealed year‐round occupation of the northern Bohai Sea and Yellow Sea coastal zones, intertidal areas, shallow waters, and the Bohai Strait (Zhuang et al. 2023). Seasonal variations show partial populations reaching South Korea's Baengnyeong Island, the Korea Strait, and the Sea of Japan during summer months, while a resident subgroup persists in the Bohai Sea or migrates southward to the Yangtze River estuary and Fujian coastal waters (Won and Yoo 2004; Yan et al. 2018). High‐resolution monthly observations reveal intricate migration patterns: During March and April, spotted seals inhabiting the Shandong Peninsula migrate to the Bohai Sea, while those in Liaodong Bay split into two groups, one migrating to the Shandong Peninsula and the other to the eastern Liaodong Peninsula. From May to June, Liaodong Peninsula populations relocate to the Korean Peninsula, while Bohai subgroups disperse to both the Korean Peninsula and Yangtze River estuary. Between July and August, Korean Peninsula populations predominantly return to the Liaodong Peninsula, concurrent with partial Yellow Sea groups migrating to the Shandong Peninsula. By September, Liaodong Peninsula aggregations initiate congregation in Liaodong Bay, while Shandong Peninsula subgroups exhibit bifurcated behavior, some migrating to Liaodong Bay and others remaining locally. Notably, the southern Bohai Strait emerges as a critical ecological corridor across all migratory pathways (Zhuang et al. 2023). This intricate spatial–temporal pattern highlights the species' behavioral plasticity in response to seasonal environmental dynamics and resource availability.

## 3 Molecular Studies on Population Genetics

Current research on spotted seals predominantly focuses on field investigations of population distribution, behavioral ecology, and captive breeding, while understanding of population structure and demographic status remains constrained. DNA markers and sequencing technologies enable the detection of nucleotide variations, thereby facilitating investigations into species' genetic diversity. Compared to conventional field surveys, molecular‐level studies on spotted seals have been relatively limited. Existing molecular biological research primarily employs PCR‐based marker systems, including microsatellite DNA, amplified fragment length polymorphism (AFLP), and major histocompatibility complex (MHC) analyses, alongside DNA sequencing‐based approaches such as mitochondrial DNA (mtDNA) profiling. Recent years have witnessed preliminary explorations into spotted seal genomics, transcriptomics, and proteomics, expanding the methodological framework for understanding their adaptive evolution and population dynamics at molecular scales.

### 3.1 PCR‐Based Molecular Markers

Microsatellite DNA, characterized by simple sequence repeats (SSRs) of 1–5 nucleotide units, is ubiquitously distributed across eukaryotic genomes. These loci exhibit polymorphism across individuals and populations through variable allele numbers at identical microsatellite sites, enabling genetic distance estimation among groups. This marker system has been extensively applied in population genetic structure analysis, evolutionary history reconstruction, and genetic mapping. A study employing 29 microsatellite primers to analyze 176 spotted seals from the Liaodong Bay ice zone, Dalian coastal waters (Liaoning), and Yantai coastal waters (Shandong) found no significant differences in genic or genotypic frequencies among these groups, indicating their membership in a single metapopulation (Han et al. 2010). AFLP, a technique utilizing primers with randomized nucleotide variations to amplify restriction‐digested genomic DNA fragments, detects polymorphism through differential amplification patterns. Analysis of 43 spotted seals over 3 years using seven AFLP primer pairs revealed low genetic diversity and simplified genetic structure in the Liaodong Bay population (Hao et al. 2008; Sun 2009).

MHC, a hypervariable gene family central to vertebrate immune function, correlates with population viability, reproductive fitness, pollutant resistance, and pathogen defense, serving as a proxy for genome‐wide variation (Ting and Qian 2011). Reduced MHC diversity increases susceptibility to emergent pathogens and compromises survival capacity. Investigations of MHC‐I and MHCII‐DQB exon 2 polymorphisms in spotted seals identified substantial allelic richness, suggesting retained high MHC diversity (Han 2009; Ting and Qian 2011). This may reflect adaptive responses to escalating marine pathogen diversity driven by environmental pollution and habitat degradation, where elevated MHC variability enhances environmental adaptability under intensified selection pressures.

### 3.2 DNA Sequencing‐Based Markers

mtDNA has rapid evolutionary rates, strict maternal inheritance, and minimal recombination; therefore, it has been served as a critical molecular tool for investigating population genetic structures (Han 2009) (Table 1). The mtDNA genome comprises coding and non‐coding regions, with the coding region in seals containing 37 genes: 22 tRNA genes, 2 rRNA genes, and 13 protein‐coding genes. Arnason et al. (2006) first sequenced the complete mtDNA of a spotted seal while reconstructing pinniped phylogeny. The non‐coding control region (D‐loop region), located between tRNA^thr^ and tRNA^phe^, harbors essential sequences for DNA replication and transcription. As the most evolutionarily dynamic and polymorphic segment of mtDNA, it has become a pivotal marker for evolutionary studies (Gao et al. 2009).

**TABLE 1:** Commonly used primers for mitochondrial DNA (mtDNA) amplification in spotted seals population studies.

Amplicon region	Primers (5′ to 3′)	References
A portion of the threonine and proline tRNA genes and part of the control region (~750–1000 bp)	L16274_F:TACACTGGTCTTGTAAACC H34_R:CCAAATGCATGACACCACAG	Li (2007), Li et al. (2010), Q. Wang (2006)
A portion of the threonine and proline tRNA genes and part of the control region (~750–1000 bp) and ND4L gene (~600 bp)	L16274_F:TACACTGGTCTTGTAAACC H34_R:CCAAATGCATGACACCACAG ND4L_F:CTTCCATGAGCATCGCACACAGA ND4L_R:GGCTTATGCAATTGTCACCGAGT	Liu et al. (2022)
The full length of mtDNA (16,754 bp)	L16274_F:TACACTGGTCTTGTAAACC COI_R:ACGATGTGTGAGATTATTCCG COI_F:GGCACTCTTTATTTGCTGTTT ND4L_R:GGCTTATGCAATTGTCACCGAGT ND4L_F:CTTCCATGAGCATCGCACACAGA H34_R:CCAAATGCATGACACCACAG	Gao et al. (2017)
Upstream of the control region CSB‐3 (~1000 bp)	DL_F:GACACAACTCTCCCTAAGAC DL_R:CCTTTGTGTTTATGGAGCCT	Sun (2009)

Multiple analyses based on mtDNA control region consistently demonstrate genetic homogeneity among populations along China's Liaoning and Shandong coasts and South Korea's western coast, confirming migration among these regions (Li 2007; Q. Wang 2006). Whole mitochondrial genome sequencing via Long and Accurate Polymerase Chain Reaction (LA‐PCR) combined with primer walking strategies yielded congruent results (Gao et al. 2017). Notably, the Liaodong Bay population exhibited markedly lower genetic diversity compared to groups in the Sea of Japan, Sea of Okhotsk, and Alaskan waters (Han et al. 2007; Li et al. 2010; Q. Wang 2006). This depletion may be attributed to historical overhunting‐triggered population bottlenecks during the last century, compounded by genetic drift effects (Han 2009). Phylogenetic reconstructions suggested the Liaodong Bay population may originate from Japanese group dispersal, with historical gene flow between these now genetically divergent populations. Conversely, analyses of the CSB‐3 upstream region in the control region revealed substantial DNA heterogeneity and polymorphism levels, though the underlying mechanisms remain unclear (Sun 2009). Analyses of control region amplification have demonstrated elevated genetic diversity in the current Liaodong Bay spotted seal population compared to levels observed 15 years prior, molecularly validating the conservation efficacy of protective measures implemented over recent decades (Liu et al. 2022).

### 3.3 Genomic, Transcriptomic, and Proteomic Studies

The prolonged low genetic diversity in Chinese spotted seal populations limits the utility of traditional neutral markers (microsatellites, mtDNA) for assessing genetic flow and adaption. The recent development of multi‐omics technologies has provided us with the possibility to analyze the adaptive evolutionary mechanism of the spotted seals. Whole‐genome sequencing and genomic analyses were conducted on one male and one female spotted seal from China's Liaodong Bay in 2019, identifying and characterizing molecular markers including microsatellites and single nucleotide polymorphisms (SNPs) (Xu et al. 2024). Pioneering transcriptomic sequencing by Gao et al. (2013) revealed 193 defense‐related genes, 20 MHC‐associated genes, and thousands of microsatellite sequences in spotted seals. Label‐free proteomic profiling of blood samples from wild and captive pups identified 972 proteins functionally linked to metabolic, immune, and cellular processes, with 51 exhibiting significant expression differences between groups (Tian, Du, Han, Bao, et al. 2020). Heat shock protein 90‐β emerged as a central indicator among differentially expressed proteins, marking a key divergence between wild and captive individuals. Subsequent isobaric tagging‐based quantitative proteomics further delineated expression variances: 158 proteins differed between wild and short‐term captive seals, 140 between short‐ and long‐term captive groups, and 235 between wild and long‐term captive populations (Tian, Wang, et al. 2022). Wild pups demonstrated elevated immune‐related protein levels, likely reflecting heightened survival pressures compared to captive counterparts. These multi‐omics datasets provide fundamental resources for advancing genetic, evolutionary, and conservation studies of Chinese spotted seal populations.

Multiple factors can drive biodiversity loss (i.e., genetic, species, and ecosystem diversity), including climate change, pollution, and notably, habitat degradation, fragmentation, and destruction. Genetic diversity is compromised earlier than species diversity, underscoring the critical need to investigate genetic diversity dynamics under global change scenarios. Integrating landscape genomics frameworks with multiple environmental gradients (e.g., rising temperatures, pollutant levels) and multi‐omic characteristics (genomic, transcriptomic, proteomic) not only enhances understanding of genetic patterns across temporal and spatial scales but also correlates landscape heterogeneity with estimates of gene flow and functional connectivity. This functional connectivity is paramount for tracking shifting ecological niches under climate change (Pauls et al. 2013). Furthermore, by identifying adaptive loci associated with environmental changes such as pollutant metabolism and thermotolerance, it becomes possible to examine the potential dispersal of adaptive genes across landscapes (Manel and Holderegger 2013). Ultimately, this strategy is expected to be able to forecast the future spatial distribution of adaptively relevant genetic variation across species' entire ranges under combined climatic and anthropogenic pressures.

## 4 Gut Microbiome of Spotted Seals

**FIGURE 2:**
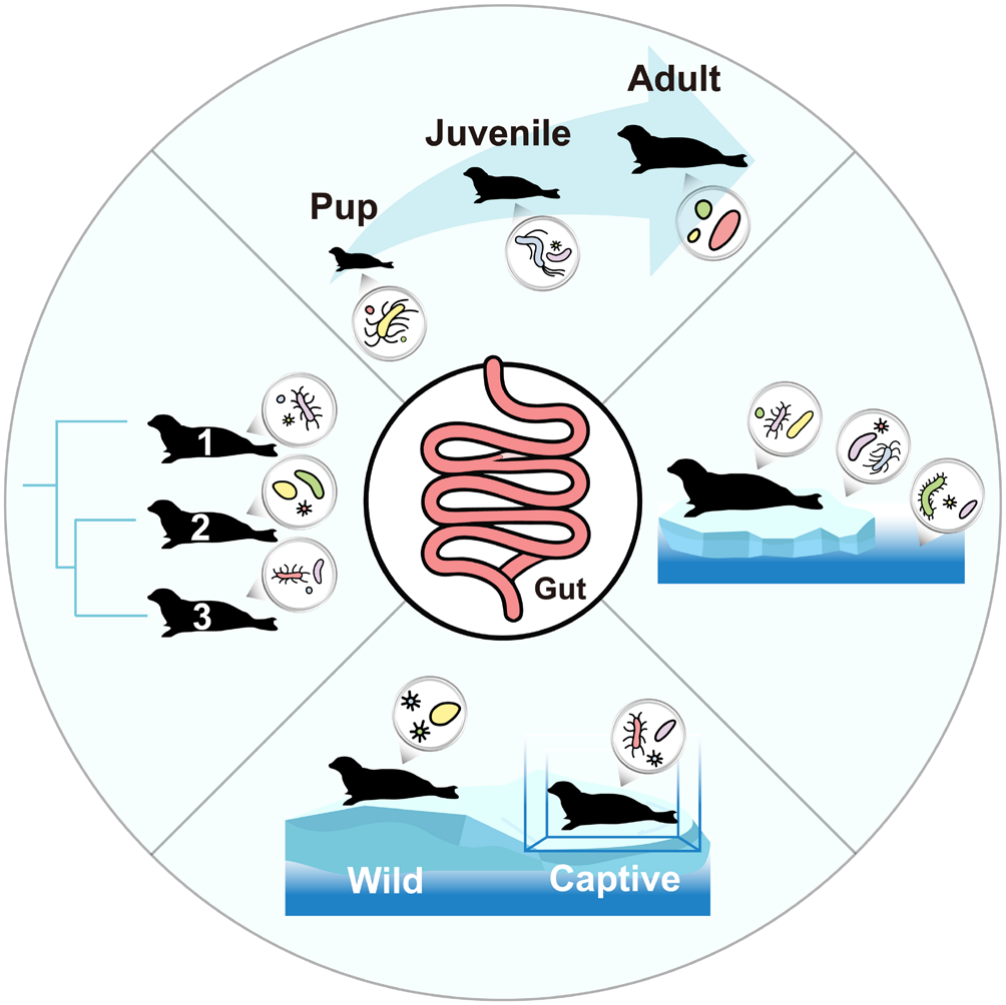
A conceptual framework of key research priorities for the spotted seal gut microbiome.

Marine mammals, as long‐lived apex predators, serve as sentinel species for both oceanic and human health. Multiple anthropogenic and environmental stressors, including overfishing, habitat degradation, marine pollution, climate change, and infectious disease outbreaks pose significant threats to these organisms. Investigating marine mammal health provides critical insights into marine ecosystem responses to environmental perturbations (Tian, Sanganyado, et al. 2022). The gut microbiome, a complex microbial ecosystem within the digestive tract, plays vital roles in host food digestion, nutrient assimilation, immune regulation, and overall health, earning its designation as the “second genome” (Zhu et al. 2010). Pinnipeds (e.g., seals), characterized by piscivorous diets rich in proteins and unsaturated fatty acids, exhibit distinct gut microbial profiles compared to other marine organisms and terrestrial carnivores. Furthermore, interindividual variations in gut microbiome composition arise from dietary differences, geographic distribution, and host genetic factors (Tian, Du, Han, Song, and Lu 2020). Therefore, understanding the gut microbial communities holds critical importance for elucidating the health status, feeding ecology, genetic diversity, and conservation strategies of pinnipeds. As fecal microbiomes constitute non‐invasive indicators of host health status (Wu et al. 2024), current research strategies predominantly employ fecal DNA extraction and microbial diversity analysis to investigate the spotted seal gut microbiome (Dong et al. 2025). For the past few years, key research priorities include: (1) characterizing composition and successional dynamics of the spotted seal gut microbiome; (2) elucidating relationships between gut microbial communities and habitat‐associated microbiomes; (3) examining age‐dependent microbial succession patterns; and (4) comparing wild and captive populations to assess captivity‐induced microbiome alterations and associated health impacts (Figure 2). Systematic monitoring of gut microbiome provides valuable biomarkers for evaluating marine mammal health and formulating evidence‐based conservation strategies for endangered species (Table 2).

**TABLE 2:** Case studies on the gut microbiome of spotted seals.

Time	Age	Sample information	Technique	References
—	Pup (< 1 year old), juvenile (1–2 years old), subadult (2–3 years old), and adult (≥ 4 years old)	Six fecal samples were collected in each age class, totally 24 samples	Sequencing of the V3 − V4 region of the 16S rRNA gene	Tian, Du, Han, Song, and Lu (2020)
2019.03	Pup	Three fecal samples were collected every 3 days, totally 27 samples	Sequencing of the V3 − V4 region of the 16S rRNA gene	Tian, Du, Han, Wang, et al. (2020)
2021.01	—	Fecal samples were collected from sea ices and surrounding water in three locations, totally 14 samples	Sequencing of the V3 − V4 region of the 16S rRNA gene	Tian, Sanganyado, et al. (2022)
2022.03–05	Adult	Fecal samples from wild (18) and captive (20) spotted seals	Full‐length PacBio sequencing of the 16S rRNA gene	Du, Wang, Gao, et al. (2024); Du, Wang, Xing, et al. (2024)
2023.03	Adult	Surface water samples (8), sediment samples (7), and fecal samples (27) from wild spotted seals	Full‐length PacBio sequencing of the 16S rRNA gene	Du, Wang, Gao, et al. (2024)
2021.09–2022.08	Adult	Monthly sampling of three captive spotted seals, totally 36 samples	Sequencing of 16S rRNA gene	Liu et al. (2024)
2021.01, 2022.01	Adult	Fecal samples from wild (18) and captive (20) spotted seals	Metagenomic sequencing	Wang et al. (2024)
2021.05	Adult and juvenile	Fecal samples from captive spotted seals (24)	Sequencing of the V3 − V4 region of the 16S rRNA gene	Dong et al. (2025)

Spotted seals exhibited elevated α and β diversity in their gut microbiome during spring and summer (Liu et al. 2024). Comparative studies across pinniped species identified Firmicutes as the dominant phylum, with multiple investigations confirming Firmicutes and Proteobacteria as predominant taxa in spotted seal guts, demonstrating seasonal fluctuations (Liu et al. 2024; Tian, Du, Han, Song, and Lu 2020; Tian, Du, Han, Wang, et al. 2020; Wang et al. 2024). The relative abundance of Firmicutes was lowest during summer and comparatively higher in winter, suggesting that the synergistic interaction between low‐temperature environments and winter‐specific dietary habits may constitute a key regulatory factor driving seasonal variations in microbial composition (Liu et al. 2024). Given the species' high adiposity, Firmicutes may regulate lipid metabolism and enhance energy harvest (Tian, Du, Han, Song, and Lu 2020). Comparative analyses between spotted seal gut microbiomes and habitat microbiomes (i.e., seawater and sea ice) revealed reduced microbial diversity in seals, accompanied by simpler yet distinctive co‐occurrence networks in gut communities (Du, Wang, Gao, et al. 2024). Certain gut microbes potentially originated from sea ice, exhibiting antibiotic resistance and pathogenic capabilities (Tian, Sanganyado, et al. 2022). Significant enrichment of potential pathogens and human disease‐associated bacteria in guts not only indicates population health risks but also suggests their role as pollution vectors exacerbating ecosystem‐wide health threats.

Age‐dependent microbial variations emerged across developmental stages (< 1 year, 1–2 years, 2–3 years, ≥ 4 years), with 2 and 4 years marking critical transition points in microbial community structure (Tian, Du, Han, Song, and Lu 2020). Adult spotted seals harbored more diverse microbes with complex, stable microbial interactions. *Clostridium sensu stricto 1* was identified as a dominant and prevalent genus across all age groups, comprising up to 17% and 15% of the microbial community in adults and juveniles, respectively (Dong et al. 2025). *Clostridium* species, prevalent in marine mammal guts, benefit intestinal homeostasis through anti‐inflammatory mechanisms via epithelial cell activation, barrier enhancement, and immune modulation (Guo et al. 2020). Notably, the pathogenic *Clostridium perfringens* persisted in both juvenile and adult seals (Dong et al. 2025; Tian, Du, Han, Wang, et al. 2020). Functional predictions indicated transporter pathway enrichment in juveniles versus ribosomal pathway dominance in adults (Dong et al. 2025).

Captive and wild populations demonstrate marked divergence in microbial diversity, composition, and functionality. Compared with wild seals, captive seals exhibited monthly microbial shifts under stable conditions, with elevated diversity characterized by increased Firmicutes and reduced Proteobacteria abundances (Du, Wang, Xing, et al. 2024; Liu et al. 2024; Wang et al. 2024). While pathogen profiles overlapped between groups, wild individuals show greater inter‐individual variability (Du, Wang, Gao, et al. 2024). Network analyses revealed enhanced complexity and stability in captive gut microbiomes, whereas wild seals maintained superior nutrient biosynthesis and innate immune capabilities (Wang et al. 2024). Captive individuals displayed attenuated stress responses but heightened carbohydrate metabolism, likely attributable to controlled diets and environments (Du, Wang, Xing, et al. 2024). Overall, current findings validate China's captive breeding strategies in improving microbial diversity and stability in spotted seal guts. Conversely, the enhanced complexity and stability observed in captive spotted seal microbiomes reflect adaptations to artificial environments. Elevated carbohydrate metabolism coupled with reduced lipid metabolism may compromise digestive efficiency for high‐lipid prey species following reintroduction to natural habitats. The captive‐adapted, simplified gut microbiome may reflect a narrowed ecological niche. This state could impair critical post‐release survival capacities, such as dietary flexibility and pathogen defense, thereby potentially reducing long‐term fitness (Mckenzie et al. 2017; Trevelline et al. 2019). However, empirical evidence directly investigating this relationship remains absent in current research. Long‐term monitoring of microbial communities in captive marine mammals is of paramount importance, as it not only facilitates the elucidation of how dietary composition, medical history, and captive environmental conditions shape their microecosystems, but also enables the optimization of animal health management protocols through dynamic tracking of microbial fluctuations. This systematic approach provides scientific substantiation for improving physiological adaptability and welfare standards in captive animals (Liu et al. 2024).

In 2022, Liaoning Province of China issued a local standard: Specification for health assessment of the gut microbiota of *Phoca largha* (DB21/T 3646‐2022), which outlines methods for collecting fecal samples from spotted seals and establishes health indicators for their gut microbiota. This pioneering standard fills a critical gap by providing the first specialized framework to scientifically evaluate gut microbiome health in this vulnerable marine species. It enables non‐invasive monitoring of spotted seal population health, informing targeted conservation strategies for both captive and wild populations. Building upon this foundation, subsequent efforts can further incorporate functional genes and metabolic pathways into standardized frameworks. Updating these protocols to include key functional metrics, such as antibiotic resistance gene abundance and short‐chain fatty acid synthesis capacity, will enhance assessments of spotted seal community health. Furthermore, as apex predators, taxonomic and functional variations within their gut microbiota constitute valuable bioindicators, providing critical insights into marine ecosystem‐wide impacts stemming from pollution or climatic perturbations.

## 5 Challenges and Future Directions

Current studies on spotted seal population genetic structure predominantly rely on limited molecular markers, including select microsatellite loci and mtDNA regions. Multiple methodologies consistently indicate historical genetic bottleneck effects and persistently low genetic diversity in the Liaodong Bay population, reflecting past environmental adversities. Around 2010, genetic diversity assessments using mtDNA and related markers have gradually decreased and stagnated, despite population recoveries in both wild and captive groups. This stagnation highlights critical knowledge gaps regarding contemporary genetic diversity restoration levels in the Liaodong Bay population. Concurrently, rapid advancements in omics technologies and reduced sequencing costs have redirected research focus toward genomic and proteomic investigations, complemented by multi‐omics approaches for gut microbiome characterization. While fecal DNA analysis remains the primary strategy for microbiome studies, the number of fecal samples in previous studies is relatively small, with a coverage rate of less than 1% for the entire population, and the representativeness of the gut microbial structure detected is largely unknown. Moreover, investigations into the source‐sink dynamics between spotted seal gut microbiomes and ambient habitat microbiomes have reported marked discrepancies across studies, with substantial uncertainty remaining in extant findings. A key factor contributing to this uncertainty is that many studies either inadequately documented sequencing depth or employed relatively low sequencing depth, casting doubt on whether observed microbial diversity profiles and compositional patterns authentically represent in situ ecological realities. Therefore, methodologically robust experimental designs addressing both sample size adequacy and sequencing depth optimization are imperative for future investigations to resolve these knowledge gaps.

**FIGURE 3:**
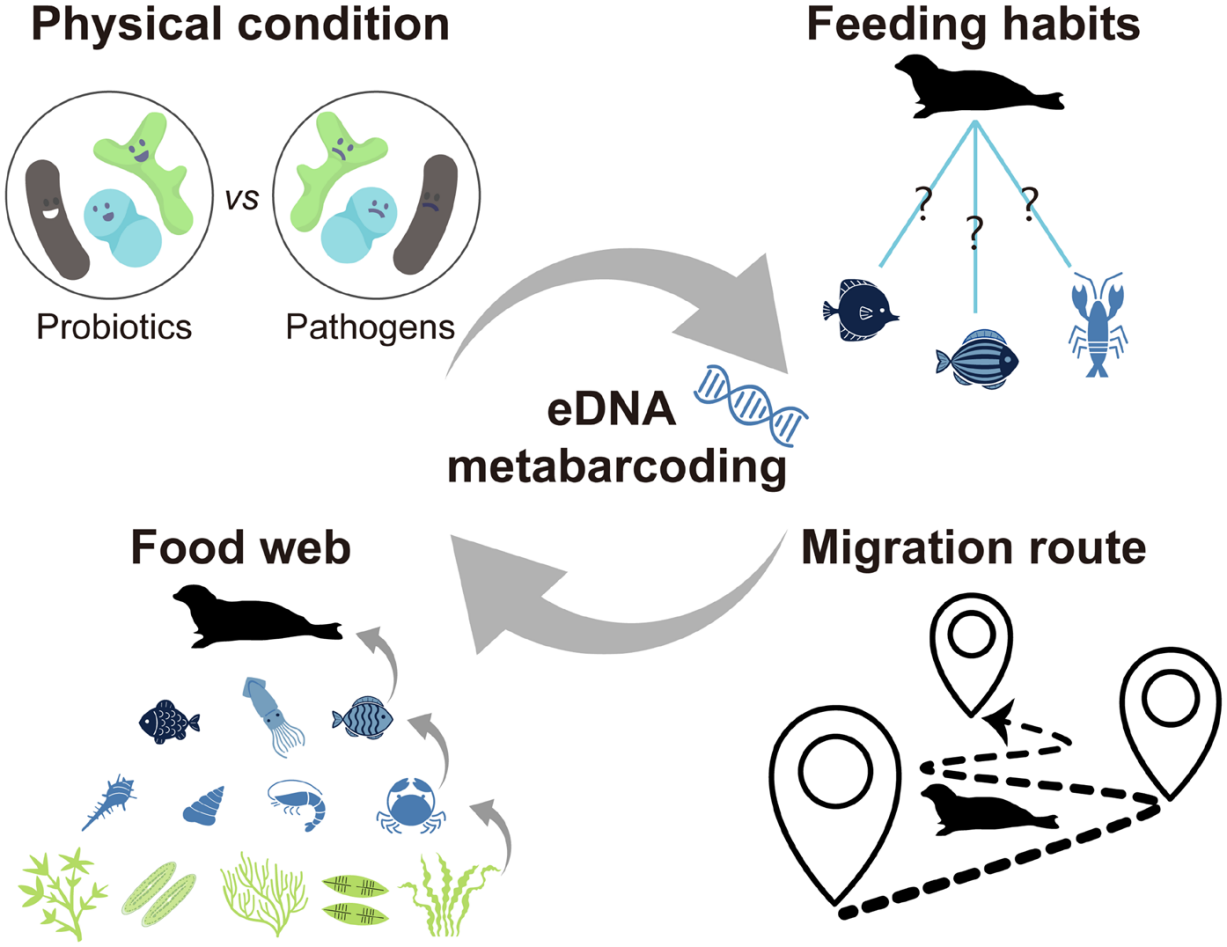
The application prospects of environmental DNA (eDNA) technology.

eDNA technology, which extracts organismal DNA directly from environmental samples (e.g., water, sediment) for high‐throughput sequencing of target genes, offers non‐invasive, cost‐effective, and sensitive monitoring of aquatic ecosystems (Altermatt et al. 2025). Although the application of eDNA in aquatic bioassessment currently faces limitations such as the rapid degradation of DNA in dynamic marine environments affecting temporal resolution, the potential for false negatives when detecting rare or low‐biomass species, and challenges in precisely distinguishing closely related sympatric species from water samples alone (Mauvisseau et al. 2022), this approach enables the simultaneous acquisition of biodiversity data across multiple trophic levels through single sampling efforts. Consequently, eDNA‐based approaches not only enable rapid reconstruction of megafaunal community structure profiles and characterization of gut microbial consortia, but also permit simultaneous analysis of trophic ecology and food web architecture. Therefore, we propose evolving eDNA technology from biodiversity censuses to multi‐trophic level detection and interaction analysis (Figure 3).

Firstly, detection of beneficial and pathogenic bacteria within spotted seals can quantify population stress levels when coupled with simultaneous profiling of environmental microbiomes and host‐pathogen interactions. This approach further enables early‐warning systems for zoonotic disease risks (Amarasiri et al. 2021). Secondly, investigations into dietary preferences and food web interactions yield direct empirical insights into the diversity‐structure–function continuum of ecological communities, while simultaneously establishing a methodological framework for developing scientifically grounded conservation strategies that address ecosystem integrity preservation (Shao et al. 2019). For example, a recent investigation employing 12S rRNA gene metabarcoding on fecal DNA of spotted seals successfully identified piscine prey spanning 5 orders, 8 families, and 13 genera, with mullets and Gobiidae constituting dominant dietary components (Gao et al. 2024). Finally, while satellite telemetry has enabled migration tracking for limited individuals, the complete migratory trajectories of the entire Liaodong Bay population remain unresolved. Novel satellite remote sensing and machine learning approaches present unprecedented opportunities for rapid and precise global biodiversity monitoring. Deep learning techniques have demonstrated capabilities in satellite‐based tracking of mammalian populations across heterogeneous landscapes (Wu et al. 2023). Integrating machine learning analysis of individual behavioral patterns from satellite imagery with high‐resolution trophic profiling via fecal eDNA metabarcoding, while supplemented by comparative analysis of biogeographic fish distribution patterns across regions, can elucidate population‐level movement ecology dynamics and further constructs foraging‐migration coupling maps. This methodological integration ultimately establishes an evidence‐based conservation framework to enhance survey efficacy and protection strategies for spotted seal populations worldwide.

## Author Contributions

**Shuzhen Li:** conceptualization (lead), formal analysis (lead), funding acquisition (lead), writing – original draft (lead), writing – review and editing (lead). **Wenzhuo Li:** investigation (equal), writing – review and editing (equal). **Xuwang Zhang:** investigation (equal), writing – review and editing (equal). **Hao Zhou:** investigation (equal), writing – review and editing (equal). **Jingjing Zhan:** investigation (lead), writing – review and editing (lead).

## Funding

This work was financially supported by the Fundamental Research Funds for the Central Universities (DUT25RC(3)070).

## Conflicts of Interest

The authors declare no conflicts of interest.

## Data Availability

Data sharing not applicable to this article as no datasets were generated or analyzed during the current study.
